# How do we estimate survival? External validation of a tool for survival estimation in patients with metastatic bone disease—decision analysis and comparison of three international patient populations

**DOI:** 10.1186/s12885-015-1396-5

**Published:** 2015-05-22

**Authors:** Andrea Piccioli, M. Silvia Spinelli, Jonathan A. Forsberg, Rikard Wedin, John H. Healey, Vincenzo Ippolito, Primo Andrea Daolio, Pietro Ruggieri, Giulio Maccauro, Alessandro Gasbarrini, Roberto Biagini, Raimondo Piana, Flavio Fazioli, Alessandro Luzzati, Alberto Di Martino, Francesco Nicolosi, Francesco Camnasio, Michele Attilio Rosa, Domenico Andrea Campanacci, Vincenzo Denaro, Rodolfo Capanna

**Affiliations:** 1 The Italian Orthopaedic Society Bone Metastasis Study Group, Via Nicola Martelli, 3, 00197 Rome, Italy; 2 Department of Molecular Medicine and Surgery, Section of Orthopaedics and Sports Medicine, Karolinska University Hospital, Karolinska Institute, Stockholm, Sweden; 3 Department of Surgery, Memorial Sloan-Kettering Cancer Center, New York, NY USA

**Keywords:** Skeletal metastasis, Prognostic model, Postoperative survival, Bayesian statistics

## Abstract

**Background:**

We recently developed a clinical decision support tool, capable of estimating the likelihood of survival at 3 and 12 months following surgery for patients with operable skeletal metastases. After making it publicly available on www.PATHFx.org, we attempted to externally validate it using independent, international data.

**Methods:**

We collected data from patients treated at 13 Italian orthopaedic oncology referral centers between 2010 and 2013, then applied to PATHFx, which generated a probability of survival at three and 12-months for each patient. We assessed accuracy using the area under the receiver-operating characteristic curve (AUC), clinical utility using Decision Curve Analysis (DCA), and compared the Italian patient data to the training set (United States) and first external validation set (Scandinavia).

**Results:**

The Italian dataset contained 287 records with at least 12 months follow-up information. The AUCs for the three-month and 12-month estimates was 0.80 and 0.77, respectively. There were missing data, including the surgeon’s estimate of survival that was missing in the majority of records. Physiologically, Italian patients were similar to patients in the training and first validation sets. However notable differences were observed in the proportion of those surviving three and 12-months, suggesting differences in referral patterns and perhaps indications for surgery.

**Conclusions:**

PATHFx was successfully validated in an Italian dataset containing missing data. This study demonstrates its broad applicability to European patients, even in centers with differing treatment philosophies from those previously studied.

## Background

Estimating survival in patients with skeletal metastases is important to set patient and physician expectations, as well as to guide surgical decision making [1–4]. During the preoperative evaluation, survival estimates can help surgeons carefully avoid under- or overtreatment of the disease by identifying which patients are likely to benefit from surgery but also whether a more durable implant may be necessary [5, 6]. Though most physicians are able to derive subjective survival estimates, they are generally inaccurate, and treating surgeons may be uncomfortable recording them in the medical record, or communicating them directly to patients [7].

With this in mind, we developed a Bayesian Belief Network capable of estimating three and twelve month survival in patients undergoing surgery for skeletal metastases [8]. It is intended to guide, not replace, good clinical judgment and uses prognostic variables previously demonstrated to risk stratify these patients, including the oncologic diagnosis [8, 9], the extent of disease [10], the patient’s performance status [11], and basic laboratory assessments [12]. In addition to delivering the likelihood of survival, PATHFx also estimates the quality of evidence supporting that estimation, which can be used by the treating surgeon to qualify each estimate. Following its development, we ensured that the tool was suitable for the clinical setting by performing decision curve analysis [13, 14], externally validated it using Scandinavian registry data [15], and made it publicly available to the international community, without charge, on www.PATHFx.org. However, the success of this tool depends on its performance in a variety of cultures, patient populations and institutions that may have differing treatment philosophies from those previously studied. In addition, though PATHFx was designed using the records of patients with metastases of the appendicular and axial skeleton, the first external validation set lacked patients treated for axial lesions. As such, additional validation studies are needed that include patients with both appendicular and axial metastases.

The purpose of this study was to (1) externally validate the PATHFx tool in an Italian patient population by evaluating accuracy by ROC analysis and clinical utility using DCA, and (2) compare the distributions of patients to both the training set (U.S.) and first external validation (Scandinavian) datasets, respectively.

## Methods

### Data collection

The Italian Society of Orthopaedic and Traumatology (SIOT) established the Bone Metastasis Study Group in order to study patients with bone metastases and improve treatment. In the current study we retrospectively reviewed the records of 287 patients from 2010 to 2013 treated at one of thirteen Italian referral centers. Each record contained the 17 demographic and clinical variables, required of the PATHFx models. Survival was defined as the time elapsed from the date of surgery to the date of death or last follow-up. All records had sufficient follow-up to establish 12-month survival. This study received local ethical approval from the Università Campus Bio-Medico di Roma (Prot:15/13 19 June 2013). Informed consent was not required prior to using de-identified registry data.

Though data was collected from multiple Italian centers, the indications for surgery were standardized. In general, patients with metastatic disease of the extremities were offered surgery to prevent or treat a pathologic fracture, according to the Mirel’s criteria [16]. The surgical indications for spine metastasis were: intractable pain, the onset of neurological deficits, caused by the compression of the myeloradicular structures of the neoplastic mass or by a pathological fracture of the vertebra, the mechanical instability of the spinal segment affected by the metastasis that causes a disabling mechanical pain and/or a neurological deficit and a failure of the previous therapy [17].

The PATHFx models are Bayesian Belief Networks comprised of ten prognostic features [8]. These include: age at the time of surgery, sex, indication for surgery (impending or completed pathologic fracture), number of bone metastases (solitary or multiple), surgeon’s estimate of survival (postoperatively, in months), presence or absence of visceral metastases, presence or absence of lymph node metastases, preoperative hemoglobin concentration (g/dL, on admission to the hospital, prior to transfusion, if applicable), absolute lymphocyte count (K/μL), and the patient’s primary oncologic diagnosis, classified into one of three groups as previously described [8]. For example, lung, gastric, and hepatocellular carcinoma and melanoma were assigned to Group 1; sarcomas and other carcinomas, Group 2; and breast, prostate, renal cell, and thyroid carcinoma, multiple myeloma, and malignant lymphoma, Group 3.

The definitions used for this study were similar to those previously described [15]. Briefly, an impending pathologic fracture was one in which the degree of bone and/or cortical disruption warranted, in the opinion of the treating surgeon, prophylactic surgical stabilization to prevent fracture. Lesions that resulted in a change in bone length, alignment, rotation, or loss of height as determined by imaging, were considered completed pathologic fractures. Biopsy-proven and/or clinically obvious metastases to organs within the chest, abdomen or brain were considered visceral metastases. Only biopsy-proven metastases to the lymph nodes were considered indicative of lymph node involvement.

### External validation

Using commercially available software (FasterAnalytics, DecisionQ Corp., Washington, DC, USA), we applied data contained in the Italian validation set to the to PATHFx, which estimated the likelihood of postoperative survival at three and 12 months, for each record. We then performed Receiver-operating characteristic (ROC) curve analysis and calculated the area under the ROC curve (AUC) as a measure of accuracy. The models were used “as-is” and were not re-fit or otherwise improved using either the Scandinavian or Italian validation sets. Bayesian Belief Networks retain functionality in the presence of missing data so no other imputation methods were employed. Validation was considered successful if the AUC was greater than 0.70 and was determined a priori. We chose this threshold because the authors consider it to be the lowest acceptable limit, however, Decision Curve Analysis (DCA) [13] was performed to determine whether the models should be used clinically.

The characteristics of the Italian set were compared to those of the training set and first external validation set. Continuous variables were tabulated and presented as mean (standard deviation), median (interquartile range) and categorical variables as number (%) (Table 1). The distribution of each continuous variable was compared with the normal distribution using the Shapiro-Wilk test. Equality of variance for continuous variables was determined using the Brown-Forsythe and Levene test. Statistical differences between continuous variables versus the bivariate outcome variables were evaluated using the Mann–Whitney *U*-test and the post hoc Tukey-Kramer assessment. Categorical variables were also tabulated and associations compared using Fisher’s exact test or chi-square analysis, depending on the number of expected values in the contingency matrix. A two-tailed α of 0.05 was considered statistically significant. We used JMP® Version 9.0.2 (SAS Institute, Inc, Cary, NC, USA) and R© Version 3.0.2 (R Foundation for Statistical Computing, Vienna, Austria) for all statistical estimations.

**Table 1:** Comparison of continuous features for training (U.S.), first validation (Scandinavian) and second validation (Italian) datasets

		Training set *n* = 189	Scandinavian set *n* = 815		Italian set *n* = 287			
Feature				% missing		% missing	vs. Training set *p*	vs. Scandinavian set *p*
Age at surgery (years)^b^	Mean	62.4	66.3	0	63.1	0	0.54	<0.005*
	SD	13.7	12.8		11.7			
	Median	62.7	67		64			
	IQR	54.4, 72.2	58, 76		56, 72			
Hemoglobin concentration (mg/dL) ^a,b^	Mean	11.5	11.5	0.6	11.5	10	0.83	0.90
	SD	1.9	3.5		1.4			
	Median	11.4	11.3		12			
	IQR	10.1, 12.9	10.3, 12.6		11,13			
Absolute lymphocyte count (K/μL)^a,b^	Mean	1.2	1.2	83.8	1.3	23	0.59	0.40
	SD	1.3	0.74		0.50			
	Median	1.0	1.2		1.5			
	IQR	0.6, 1.5	0.8, 1.6		1.0, 2.0			
Senior surgeon’s estimate of survival (months)^a,b^	Mean	10.3	N/A	100	11.2	87	0.56	N/A
	SD	8.6			7.0			
	Median	6.0			10			
	IQR	4.0, 12.0			5, 20			

*SD* standard deviation, *IQR* interquartile range, *N/A* not applicable

^*^Distributions are significantly different between training and validation sets by two-tailed Student’s *t*-test

^a^Denotes feature of 3-month model

^b^Denotes feature of 12-month model

## Results

Two-hundred eighty seven (287) records had adequate follow-up information to establish survival at 3 and 12 months postoperatively and thus comprised the validation set. None of these records were excluded.

PATHFx correctly classified three-month survival in 253 of 287 (88 %) patients, and 12-month survival in 199 of 287 (69 %) patients. On ROC curve analysis, the AUCs were 0.80 and 0.77, respectively, for the three and 12-month survival, respectively.

**Fig. 1:**
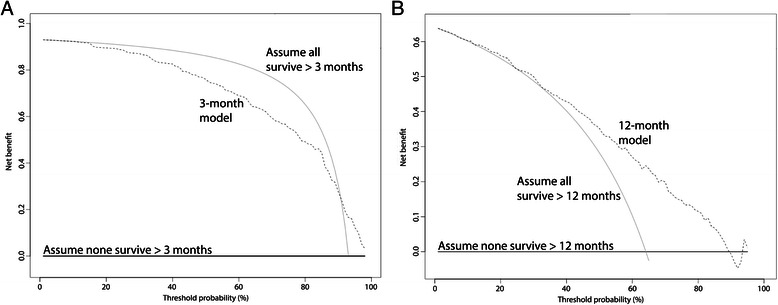
These decision curves depict the net benefit of the three-month (**a**) and 12-month (**b**) models, when applied to the Italian external validation set. Net benefit is defined as a three- or 12-month survivor who duly undergoes surgery, or receives an implant commensurate with his/her estimated survival. It is important to note that nearly all (93 %) patients referred for orthopaedic intervention survived longer than three months and 63 % survived longer than one year, representing the theoretical maximum net benefit for **a** and **b**, respectively. As a result, **a** indicates that one could achieve better outcomes by assuming all patients will survive greater than 3 months rather than using the three-month model. This analysis highlights the importance of decision analysis, even for relatively accurate models such as this one, with an AUC of 0.80. **b** indicates that the 12-month model should be used, rather than assume all patients, or none of the patients will survive greater than 12 months

Decision analysis revealed that PATHFx should be used, rather than assume all patients or no patients would ultimately survive longer than 12 months. However, since 93 % of Italian patients survived longer than three months, DCA indicated that outcomes may be better if orthopaedic surgeons assumed all patients would survive three months, rather than use the three month model (Fig. 1).

As expected, the demographic and clinical features of patients in the validation set differed from those of patients in the U.S. training set. Several features differed significantly (*p* < 0.05) including, presence of visceral and lymph node metastases, number of bone metastases, and three and 12-month survival. Nonsignificant differences were observed in age at surgery, gender, preoperative hemoglobin concentration, absolute lymphocyte count, oncologic diagnosis grouping, pathologic fracture status, ECOG performance status, and the surgeon’s estimate of survival. When compared to the Scandinavian set, most features differed significantly (*p* < 0.05) with the exception of gender, preoperative hemoglobin concentration, absolute lymphocyte count and the presence of visceral metastases. Most features in the validation set had some degree of missing data, also summarized in Tables 1 and 2. Notable features included the surgeon’s estimate of survival (missing in 87 %), absolute lymphocyte count (missing in 23 %), and ECOG performance (missing in 20 %), all of which are important first- or second-degree predictors of survival in the PATHFx tool.

**Table 2:** Comparison of categorical features for training (U.S.), first validation (Scandinavian) and second validation (Italian) datasets

Feature		Training set *n* = 189		Scandinavian set *n* = 815			Italian set *n* = 287				
		No.	%	No.	%	% missing	No.	%	% missing	vs. training set *p*	vs. Scandinavian set *p*
Gender	male	85	45.0	369	45.3	0	120	42	0	0.50	0.31
	female	104	55.0	446	54.7		167	58			
Oncologic diagnosis grouping	1.0	52	27.3	173	21.3	0.4	63	23	2	0.42	0.007*
	2.0	34	18.2	74	9.2		44	16			
	3.0	103	54.5	567	69.1		173	62			
Organ metastases	yes	114	60.3	325	39.8	6.3	91	36	12	0.0001*	0.08
	no	75	39.7	441	53.9		161	64			
Lymph node metastases	yes	36	18.8	169	20.8	61.6	96	40	16	0.0001*	0.0007*
	no	153	81.2	143	17.6		146	60			
Skeletal metastases	solitary	55	29.0	123	15.2	3.4	139	49	1	0.0001*	0.0001*
	multiple	134	71.0	666	81.4		144	51			
Pathologic fracture status	yes	84	44.2	614	75	0.9	143	52	5	0.08	0.0001*
	no	105	55.8	196	24.1		131	48			
ECOG performance status	0,1,2	93	49.2	558	68.3	0	123	54	20	0.39	0.0001*
	3,4	96	50.8	257	31.7		106	46			
Survival >3 months	yes	129	68.3	557	68.2	0	267	93	0	0.0001*	0.0001*
	no	60	31.7	258	31.8		20	7			
Survival >12 months	yes	79	41.8	241	29.8	0	181	63	0	0.0001*	0.0001*
	no	110	58.2	574	70.2		106	37			

*Abbreviations*: *ECOG* Eastern Cooperative Oncology Group, % missing, the proportion of unknown or missing data within the validation set

*Proportions are significantly different between training and validation sets by Chi-square method

## Discussion

We successfully externally validated PATHFx in an Italian patient population including patients with both axial and appendicular metastases. In doing so, we confirm the model’s ability to estimate the likelihood of survival at two time points useful for orthopaedic surgical decision-making. This is the second external validation study and demonstrates the model is also generalizable to the Italian patient population.

When one considers the goals of treating patients with skeletal metastases are to relieve pain and to restore function for the maximum amount of time, careful estimates of survival, such as those provided by PATHFx, are necessary to avoid over- or undertreatment of the disease. For example, if a surgeon considers nonoperative treatment, a very low probability of survival at three months may support this decision. By extension, if a surgeon were to consider using a less invasive and less durable implant such as an intramedullary nail, longer estimates of survival such as 3–12 months would support this decision. Conversely, estimates of survival greater than one year may support the decision to use a more durable implant such as a prosthesis in the case of extremity tumors, or more complicated spine procedures including vertebrectomies and combined anterior and posterior techniques. In fact, this study represents the first external validation of PATHFx in patients with axial (*n* = 34, 12 %), as well as appendicular skeletal metastases. This is important because although the training set contained 33 (18 %) spine patients, the Scandinavian external validation set contained only patients with extremity metastases.

Though there are several prognostic scoring systems designed for spine patients [10, 11, 18], none provide the surgeon with an estimation of the likelihood of survival at three and 12 months, which the authors consider to be useful for surgical decision-making. In addition, a recent analysis of seven prognostic tools demonstrated the Modified Bauer method [10] to be most reliable [19]. PATHFx codifies the presence of visceral metastases, number of skeletal metastases and diagnosis grouping, which are all used by the modified Bauer method, and may explain why both models function accurately, in this setting. Nevertheless, it may be important to consider neurologic impairment in patients with spine metastases, as recommended by Tokuhashi [11]. However, ECOG performance status which is also used by PATHFx may be an acceptable surrogate, since it is prognostic in patients with both axial [11] and appendicular metastases [8] notwithstanding the obvious differences in impairment due to neurologic as opposed to end-stage metastatic bone involvement. Still, we recognize the importance of a tool useful in the treatment of all patients with skeletal metastases—not simply those with spine or appendicular involvement. As such, the performance or applicability of PATHFx in the present validation set is encouraging.

PATHFx performed well in the Italian patient population, despite significant differences when compared to the training and previous validation sets (Tables 1 and 2). Importantly, 93 % of Italian patients survived longer than 3 months, which is much higher than either of the two previously studied groups. This may represent a key difference in the Italian patients, or more likely treatment philosophy and patient selection when compared to the U.S. and Scandinavian centers.

In addition, 63 % of Italian patients survived more than 12 months. This is twice as many as in the Scandinavian validation set, and nearly twice that observed in the training set. This, too may indicate key differences in patient selection and is surprising since there was a similar proportions of patients with pathologic fractures (*p* = 0.08), more-favorable diagnosis group (Group 3) (*p* = 0.42) and good performance status (ECOG 0,1,2) (*p* = 0.39) when compared to the training set. Still this may be explained by referral patterns among the Italian centers. Italian oncologists typically refer patients with excellent prognoses for orthopaedic consultation. In patients with more extensive disease and less favorable prognoses, however, surgery may be deemed unsuitable in the eyes of the oncologist, which obviates the need for an orthopaedic opinion. However, this practice may exclude patients that may benefit from less invasive stabilization or palliative procedures [20–22].

Nearly half of Italian patients included in this study presented with a solitary skeletal metastasis. This was unexpected, given that this proportion is much higher than both the training and previous validation sets, and could represent more effective disease surveillance practices than those in Scandinavia or the U.S. However, given the differences in referral patterns discussed above, it is more likely that Italian patients with less favorable prognoses—especially in the setting of impending pathologic fractures—were not referred for surgical management.

The accuracy for the three and 12-month models was 0.80 and 0.77, respectively. When compared to the original cross-validation AUCs of 0.86 and 0.83 [8], this represents a non-trivial, but acceptable 0.06-point degradation in model accuracy and is similar to that observed following external validation in the Scandinavian set (0.79 and 0.76, respectively) [15]. Still by maintaining accuracy in differing patient populations, we believe PATHFx is sufficiently robust, and DCA suggests it may be used clinically, while undergoing additional external validation in more diverse patient populations.

The PATHFx models were designed to help surgeons avoid overtreatment or undertreatment of skeletal metastases. Previous work demonstrated that the models were suitable for clinical use, and that overly optimistic or pessimistic estimates generated by PATHFx were of unequal clinical significance [14]. This is perhaps most important in the three month model that was designed to help surgeons identify which patients may benefit from a surgical or nonsurgical course of treatment. The present study demonstrated 34 (12 %) of records were misclassified by the three-month model. Of these, survival was overestimated in 13 (5 %) records, representing the maximum number of potentially unnecessary surgeries performed at the end of life. However, this estimate should be considered the theoretical maximum, since it likely includes patients who met surgical criteria and died of complications unrelated to the progression of disease. These results are more accurate than those observed in the Scandinavian set in which three month survival was overestimated in 15 % of records [15] and may be due to the larger proportion of Italian patients who survived greater than three months. If we consider that between 6 and 23 % of patients die within six weeks of surgery [23–25], then the clinical impact of such overestimates may fall within the acceptable norm.

Though one may consider an AUC of 0.8 for the three-month model to be sufficiently accurate, decision analysis helps illustrate the clinical impact of applying the model to a population in which virtually every patient referred for orthopaedic management of metastatic bone disease survives three months. Following DCA, we observe that at threshold probabilities (the point at which surgeons become indecisive about whether to offer surgery) less than 15 %, the model is equivalent to one in which all patients are expected to survive greater than three months. At thresholds >90 %, the three-month model should result in better outcomes. However, at thresholds between 15 and 90 %, an Italian orthopaedic surgeon is better off treating patients as if all will survive more than three-months, rather than use the three-month model. In the latter case, an erroneous underestimate may prompt the surgeon to withhold surgery from one in ten patients in whom it was otherwise indicated.

By extension, the 12-month model was designed to support decisions surrounding the type of procedure, as well as implant durability required for each patient. Of the 88 records misclassified by the 12-month model, survival was underestimated in 44 (15 %) cases. This represents the theoretical maximum proportion of patients at risk for implant failure if a less durable implant were used. This is higher than that observed in the Scandinavian validation set [15], in which 12-month survival was underestimated in 7.6 % of records. Though long term follow-up data were not available for this study beyond 12 months, we expect the proportion of patients surviving greater than 24 and 36 months, to decrease considerably. This trend has been observed previously [6, 8, 15], and further decreases the theoretical number of implants at risk, over time.

One of the most salient features of PATHFx is the ability to function in the presence of missing data. This attribute is particularly important when considering the surgeon’s estimate of survival was missing in 87 % of Italian and 100 % of Scandinavian records. The models maintained their accuracy because BBNs encode the information contained within the surgeon’s estimate in terms of shared, probabilistic relationships with other features, allowing one to “export” palliative expertise into settings where it may not exist. Though caution should be used when entering the surgeon’s estimate, those who are unsure of their estimate—or experience level—may simply leave it blank. Doing so will maintain accuracy of the model, while not introducing undue bias.

This study has several limitations. First, we developed PATHFx using the records of patients who underwent orthopaedic surgery for their skeletal metastases. Thus, it may not be applicable to all patients with metastatic disease, especially those in who are treated non-operatively. Second, similar to the previous Scandinavian external validation set, the Italian patient population was relatively homogeneous. However, we sought to obtain a representative sampling of Italian patients by collecting data from thirteen centers. Additionally, it is possible that PATHFx may become more accurate, by including other features potentially associated with survival in this patient population such as alkaline phosphatase [26], N-telopeptide [27, 28], and C-Reactive protein [29], or the degree of neurologic impairment as suggested by Tokuhashi. In addition, the time points chosen for PATHFx (three and 12-months) were initially chosen by two of the authors (JAF and JHH) because they are useful for orthopaedic surgical decision-making. Based on a recent study of practice patterns [30], other time points such as one-month and six-month survival may be needed in addition to the three and 12-month estimates to help surgeons decide on an operative strategy. One and six-month models are currently under development and would allow for a direct comparison with existing tools to estimate survival in patients with axial metastases, such as the Tokuhashi method [11]. Next, the degree of experience required by surgeons to provide useful, as opposed to confounding, surgeon’s estimates is under further study, as the present study is too small to derive any meaningful information. Finally, PATHFx is a clinical decision support tool and should not supplant good clinical judgment by the treating surgeon and clinical team. Palliative surgery, by definition, can be appropriate even in patients with very short life expectancies, and low estimates of survival generated by any prognostic tool should not be used to deny these types of interventions if otherwise clinically indicated.

## Conclusions

In conclusion, we successfully validated PATHFx using an Italian dataset containing patients with axial and appendicular skeletal metastases. This is the second external validation study and demonstrates that the tool is suited for clinical use in Italy. However, the three-month model should be used with caution in an Italian population, wherein nearly all (93 %) of patients referred for orthopaedic management of skeletal metastases are likely to survive longer than three months. Prospective, multicenter validation is necessary to confirm utility in other diverse patient populations and clinical settings, over time, which will provide an opportunity to assess whether the addition of newer, potentially prognostic variables could increase accuracy.
